# Neurological complications in Takotsubo syndrome: A case of concurrent acute stroke

**DOI:** 10.21542/gcsp.2025.55

**Published:** 2025-10-31

**Authors:** Badr Abdalani, Meriam Amri, Abdellah Boucetta, Omar Moufid, R. Habbal

**Affiliations:** 1 Department of Cardiology P37, Ibn Rochd University Hospital, Casablanca, Morocco; 2 Faculty of Medicine and Pharmacy, Hassan II university of Casablanca, Casablanca, Morocco

## Abstract

Takotsubo Syndrome (TTS), or stress cardiomyopathy, manifests as acute myocardial dysfunction mimicking acute coronary syndrome, with particular clinical relevance in post-menopausal women. Its diagnosis remains challenging due to overlapping features with other cardiovascular events, despite growing recognition. We report the case of a 76-year-old post-menopausal woman without major cardiovascular risk factors who developed TTS associated with ischemic stroke following the emotional stress of her son’s death. She initially presented with chest pain, later complicated by neurological deficits including weakness and speech impairment. Laboratory testing revealed elevated troponin levels, while echocardiography demonstrated typical TTS findings, further supported by the InterTAK diagnostic score and the absence of coronary obstruction on angiography. This case emphasizes the importance of considering TTS in elderly women under acute stress, highlighting the complex interplay between TTS and ischemic stroke. A multidisciplinary evaluation is required, while further studies are needed to clarify mechanisms and optimize management.

## Introduction

Takotsubo syndrome (TTS), also known as stress cardiomyopathy or ’broken heart syndrome’, is an acute myocardial disease characterized by reversible left ventricular (LV) dysfunction^1^. This form of cardiomyopathy mimics acute myocardial ischemia in its clinical presentation—including pseudo-anginal or anginal chest pain, electrocardiographic abnormalities, and elevated cardiac biomarkers, particularly troponin—yet occurs in the absence of obstructive coronary artery disease. TTS accounts for approximately 1 to 2% of cases initially suspected to be acute coronary syndrome^2^. Moreover, this condition can be complicated by an ischemic stroke (IS), even in the absence of common predisposing factors such as atrial fibrillation (AF) or intracavitary thrombi. This case report describes a 76-year-old post-menopausal woman with no modifiable cardiovascular risk factors, who presented with TTS concurrent with IS. This case underscores the importance of recognizing this condition and implementing a multidisciplinary management approach in older women.

## Case presentation

A 76-year-old post-menopausal woman with no modifiable cardiovascular risk factors and a medical history of cholecystectomy five years prior presented to the emergency department with acute, retrosternal, constrictive, non-radiating chest pain triggered by the death of her eldest son three days earlier. She reported no dyspnea, syncope, or palpitations. Two days later, she developed intense headache without any associated vomiting, followed by sudden left arm and leg weakness, speech difficulty, and facial drooping, alongside worsening chest pain, all occurring in the context of apyrexia and anorexia.

On admission, physical examination revealed a drowsy patient (Glasgow Coma Scale score 14/15) with symmetrical, reactive pupils. Vital signs were as follows: temperature 37.1 °C, capillary blood glucose 1.05 g/L, blood pressure 110/60 mmHg, heart rate 85 beats per minute, oxygen saturation 96% on room air, and respiratory rate 18 breaths per minute. The patient was hemodynamically stable with no clinical evidence of heart failure.

Neurological examination revealed an alert patient with poor orientation who followed commands appropriately. Comprehension was preserved, though orientation to time and place was impaired. The patient exhibited acute-onset left hemiparesis and dysarthria, followed by conjugate gaze deviation to the right and left homonymous hemianopia. Speech was characterized by severe dysarthria without aphasia.

Symptoms were characterized using the National Institutes of Health Stroke Scale. Cranial nerve examination demonstrated conjugate gaze deviation to the right (NIHSS score 2), left homonymous hemianopia (NIHSS score 2), and left central facial palsy (NIHSS score 2), with preserved forehead movement and weakness of the lower face.

Motor examination demonstrated left-sided hemiparesis with muscle strength of 4/5 in the left upper extremity and 3/5 in the left lower extremity (NIHSS score 7 for combined left arm and leg motor function). Deep tendon reflexes were hyperactive on the left, with a positive Babinski sign and extensor plantar response.

Assessment of higher cortical functions revealed marked left-sided spatial neglect (NIHSS score 2) and sensory extinction (NIHSS score 1). Sensory examination was otherwise intact, including light touch, pain, and proprioception. No limb ataxia was detected.

Bedside swallow screening was abnormal, indicating high aspiration risk. Collectively, these findings were consistent with acute right hemispheric stroke involving the temporoparietal region, with a total NIHSS score of 17 at admission, reflecting moderate to severe neurological deficits affecting multiple domains including cranial nerve function, motor strength, higher cortical function, and speech.

**Figure 1.**
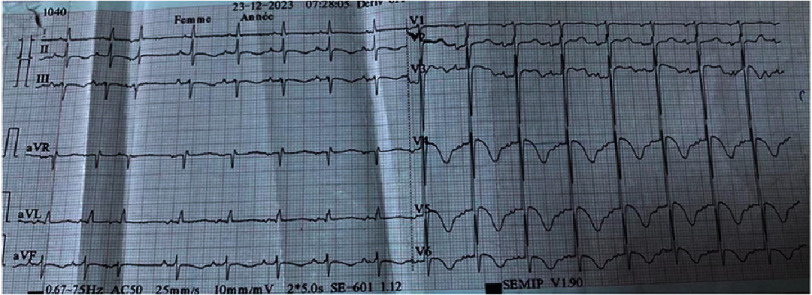
ECG at admission show a sinus rhythm, iso-electric ST segment, diffuse inverted T-waves, prolonged QTc time and a supraventricular extrasystole.

Cardiovascular and pleuropulmonary examinations were normal. An electrocardiogram (ECG) revealed regular sinus rhythm (HR 93 bpm), fixed PR interval at 160 ms, left axis deviation, iso-electric ST segment, diffuse inverted T-waves, and a QTc of 474 ms (Figure 1).

**Figure 2.**
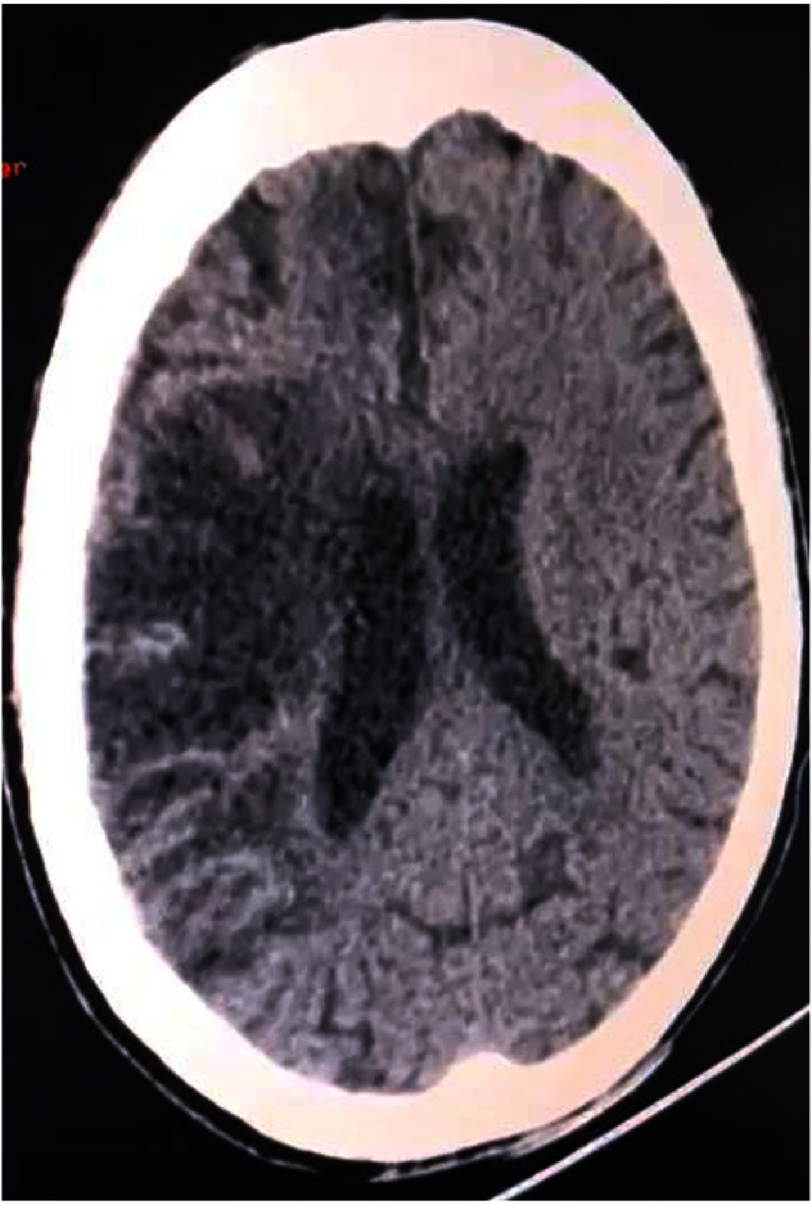
Brain CT showing a large hypodense cortico-subcortical lesion in the right MCA territory, consistent with an acute ischemic stroke, without evidence of hemorrhage.

Head CT revealed a large hypodense cortico-subcortical lesion in the right middle cerebral artery (MCA) territory with loss of gray-white matter differentiation and sulcal effacement, consistent with acute ischemic stroke. No intracranial hemorrhage or hemorrhagic transformation was identified (Figure 2).

Acute coronary syndrome (ACS), specifically non-ST-elevation myocardial infarction (NSTEMI), was suspected. High-sensitivity cardiac troponin was measured at 112.90 ng/L, with a repeat level of 61.9 ng/L three hours later. B-type natriuretic peptide (BNP) was 560 pg/mL. All other laboratory investigations were within normal limits.

**Figure 3.**
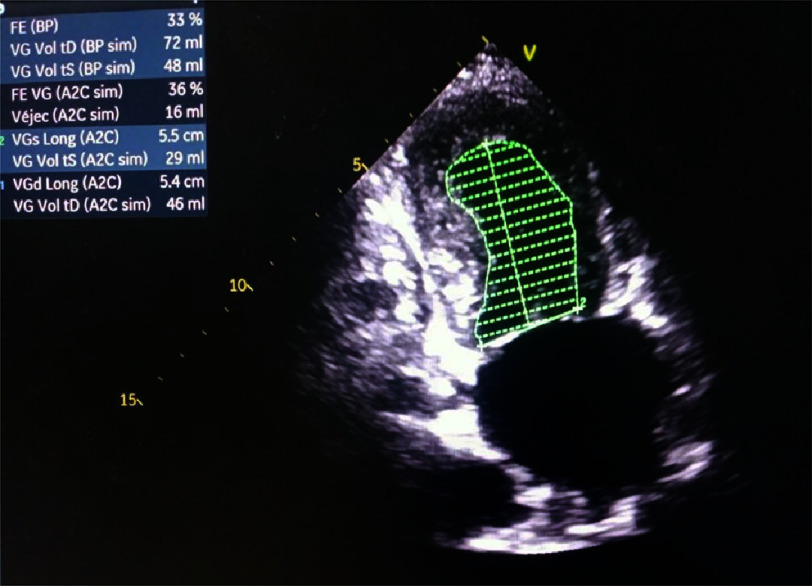
Apical two-chamber echocardiography showing left ventricular fraction at admission.

**Figure 4.**
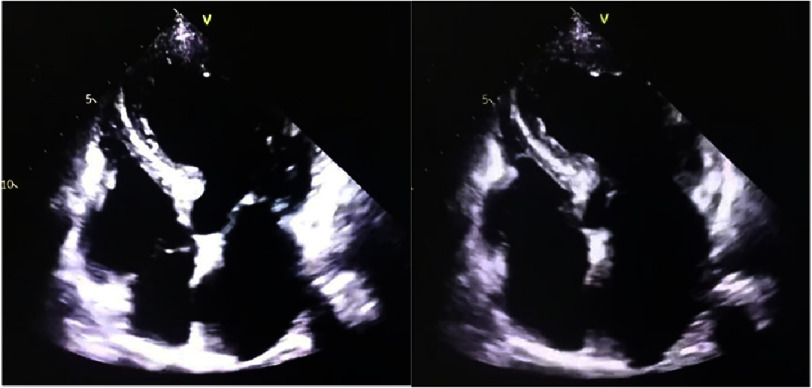
Apical four-chamber echocardiography showing the ballooning aspect of the left ventricle at admission.

**Figure 5.**
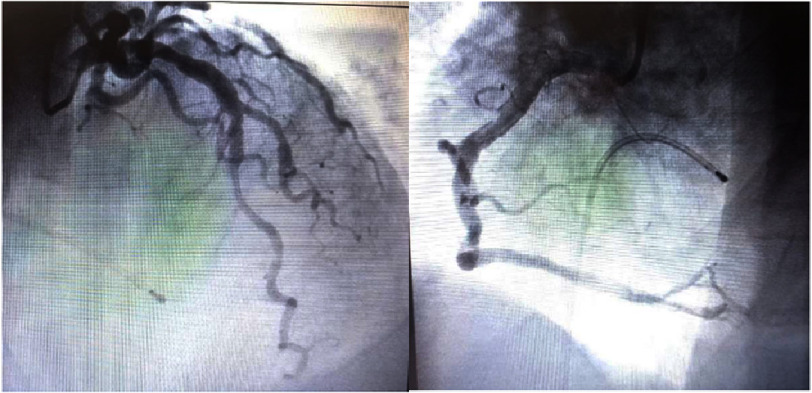
Images of the coronary angiography that were normal.

The patient was admitted to the cardiac intensive care unit with neurology consultation. Given the NIHSS score of 17, dual antiplatelet therapy was initiated with aspirin 75 mg once daily and clopidogrel 75 mg once daily, along with atorvastatin 80 mg once daily. Prophylactic enoxaparin sodium was administered for venous thromboembolism prevention in the setting of immobilization. A nasogastric tube was inserted for enteral nutrition.

Transthoracic echocardiography demonstrated apical akinesia with left ventricular ballooning and reduced ejection fraction (EF) of 33%, consistent with TTS, without evidence of intracavitary thrombus (Figures 3 and 4). Coronary angiography revealed no obstructive coronary artery disease (Figure 5). Doppler ultrasound of the supra-aortic vessels was unremarkable. TTS was confirmed based on an InterTAK diagnostic score of 89, further supported by rapid improvement in EF to 45% within 48 h. Medical management was optimized with the addition of bisoprolol 1.25 mg once daily and ramipril 1.25 mg once daily.

Transthoracic echocardiography demonstrated apical akinesia with left ventricular ballooning and reduced ejection fraction (EF) of 33%, consistent with TTS, without intracavitary thrombus (Figures 3 and 4). Coronary angiography revealed no obstructive coronary artery disease (Figure 5). Doppler ultrasound of the supra-aortic trunks was unremarkable. TTS was confirmed based on an InterTAK diagnostic score of 89, further supported by rapid improvement in EF to 45% within 48 h. Bisoprolol 1.25 mg once daily and ramipril 1.25 mg once daily were subsequently added to the treatment regimen.

The patient’s neurological status improved progressively over the first 48 h. The initial NIHSS score of 17 at admission decreased as follows: at 24 h, left-sided motor strength improved (upper extremity 4/5 → 5/5, lower extremity 3/5 → 4/5), while hemianopia and dysarthria persisted. By 48 h, level of consciousness and orientation had improved, with continued motor recovery, permitting initiation of early rehabilitation.

Multidisciplinary discussion considered whether the stroke represented a trigger or complication of TTS. Regarding therapeutic management, clopidogrel and enoxaparin were discontinued after confirming the absence of obstructive coronary artery disease and intracardiac thrombus, given the hemorrhagic risk associated with acute ischemic stroke. Aspirin, atorvastatin, bisoprolol, and ramipril were continued for cardiac recovery and secondary stroke prevention. The nasogastric tube was removed following successful swallow screening, permitting safe oral intake. Early mobilization and physical therapy were initiated to optimize functional recovery. Psychological support and family engagement were provided to address emotional distress and facilitate comprehensive recovery.

At the follow-up appointment, the patient reported no chest pain or dyspnea. ECG was normal, and transthoracic echocardiogram revealed a LVEF of 50% two weeks post-discharge. Over six months, regular follow-ups showed clinical improvement, adherence to treatment, and continued neurological rehabilitation.

## Discussion

TTS accounts for 1–3% of acute coronary syndrome cases and 0.5−0.9% of ST-segment elevation myocardial infarction presentations. The condition predominantly affects post-menopausal women, representing over 80% of cases in women aged 50 years and older, with a median age of 67 to 70 years^3–6^. The underlying cause of this marked sex disparity remains unclear. TTS has been associated with ischemic stroke, with prevalence among stroke patients approximately 20-fold higher than in the general hospitalized population, contributing to increased morbidity and hospitalization^7^.

The pathophysiology of TTS remains incompletely understood. Excessive catecholamine release may induce myocardial dysfunction through direct myocyte toxicity and inflammation, mediated by central sympathetic activation following a triggering event, with subsequent adrenergic surge from the adrenal medulla, coronary vasospasm, and microvascular dysfunction^5,8,9^. Transient myocardial ischemia secondary to atherosclerotic plaque rupture has been proposed as an alternative mechanism, though supporting evidence remains limited^5^. Multiple factors contribute to TTS susceptibility, including psychological and physical stress, genetic predisposition, and estrogen deficiency, with the majority of cases precipitated by an identifiable emotional or physical stressor^5,9–11^.

Several mechanisms may explain ischemic stroke in the setting of TTS. Cardioembolism is a primary mechanism, particularly when severe left ventricular dysfunction with extensive apical akinesia leads to intracavitary thrombus formation and subsequent embolization; approximately 5% to 8% of TTS patients develop left ventricular thrombus^12^. Atrial fibrillation, with reported prevalence ranging from 6.4% to 25.06%, represents an additional thromboembolic risk factor^13,14^. Catecholamine-induced platelet activation and endothelial dysfunction may further increase stroke risk^12^. Alternatively, ischemic stroke may result from cerebral hypoperfusion secondary to reduced cardiac output^15^. Adrenergic surge and inflammatory processes causing microvascular dysfunction, tissue hypoperfusion, and hypoxemia, as well as coronary and cerebral vasospasm, may also contribute to stroke pathogenesis. The middle cerebral artery territory is most commonly affected in TTS-related stroke^16^.

Potential mechanisms underlying ischemic stroke in TTS include cardioembolism, particularly with severe left ventricular dysfunction and extensive apical akinesia leading to intracavitary thrombus formation and embolization. Approximately 5% to 8% of TTS patients develop left ventricular thrombus^12^. Atrial fibrillation, with prevalence ranging from 6.4% to 25.06%, represents an additional stroke risk factor^13,14^. Catecholamine-mediated platelet activation and endothelial dysfunction may further increase stroke risk^12^. Ischemic stroke may also result from cerebral hypoperfusion secondary to reduced cardiac output^15^. Additionally, adrenergic surge and inflammatory processes causing microvascular dysfunction, tissue hypoperfusion with hypoxemia, and vasospasm may contribute to stroke pathogenesis. The middle cerebral artery territory is most commonly affected in TTS-related stroke^16^.

TTS exemplifies the brain-heart axis, wherein psychological stress and limbic-autonomic dysregulation predispose the myocardium to transient dysfunction. Microvascular dysfunction bridges cardiac and cerebral pathology, as it is associated with cerebral abnormalities and reduced cerebral perfusion. Sympathetic hyperactivation and catecholamine surges link transient myocardial vulnerability to cerebrovascular susceptibility through shared neuronal and microvascular pathways^1,17^. Conversely, cardiac dysfunction and thromboembolism may adversely affect cerebral outcomes, illustrating the bidirectional nature of this brain-heart interaction.

The diagnosis of TTS is established through a combination of clinical, electrocardiographic, biochemical, echocardiographic, and angiographic findings. TTS mimics acute coronary syndrome, and misdiagnosis is common, particularly as both conditions may coexist^18^. The clinical presentation typically includes chest pain, dyspnea, and syncope^10^. Complications may include pulmonary edema, cardiac arrhythmias, and rarely, cardiogenic shock or cardiac arrest^4,5^. Thromboembolic complications occur in 1–2% of cases, particularly with severe left ventricular dysfunction^4^. Male patients may present with more severe disease and higher mortality risk^19^.

Electrocardiography reveals dynamic changes that are most evident at presentation and often transient, primarily affecting the ST segment, T waves, and QTc interval. Typical findings include widespread T-wave inversion and gradual QTc prolongation, which differ from those observed following myocardial infarction^20,21^. No specific biomarker for TTS exists, and conventional cardiac biomarkers cannot reliably distinguish acute coronary syndrome from TTS; however, B-type natriuretic peptide levels are typically higher in TTS, whereas creatine kinase and troponin levels are lower^22,23^. Elevated levels of cytokines, steroid and glucocorticoid receptors, and catecholamines have been documented in TTS^24^.

Echocardiography is essential for assessing wall motion abnormalities and left ventricular ejection fraction, typically demonstrating apical ballooning with preserved or hypercontractile basal segments, and for detecting complications including left ventricular outflow tract obstruction, mitral regurgitation, and thrombus formation^25^. Cases of dobutamine stress echocardiography-induced TTS have been reported^26^. Cardiac magnetic resonance imaging typically demonstrates increased left ventricular mass, myocardial edema, apical ballooning, and absence of late gadolinium enhancement^27^.

Coronary angiography reveals normal or non-obstructive coronary arteries; left ventriculography may demonstrate apical akinesia, basal hyperkinesis, and left ventricular outflow tract obstruction in approximately 20% of cases^8^. The InterTAK diagnostic score, developed by the International Takotsubo Registry, incorporates seven clinical parameters and may help differentiate TTS from acute coronary syndrome^8,28^.

The relationship between stroke and TTS is established but incompletely understood. Stroke may serve either as a triggering event for TTS or as a thromboembolic complication of the condition. Patients with TTS have an increased risk of ischemic stroke or transient ischemic attack within 90 days, as demonstrated by the Danish National Patient Registry^29^. This elevated risk persists during the first year following TTS diagnosis, though the absolute risk remains low^12^. In the present case, stroke as the precipitating event for TTS is more likely, given the absence of intracardiac thrombus and only moderate reduction in LVEF.

TTS has been associated with additional neurological complications, including intracranial hemorrhage, status epilepticus, seizures, and neurodegenerative disorders, further supporting the brain-heart axis^30,31^.

Risk stratification for ischemic stroke in TTS is important but lacks standardization. Proposed risk indicators include mitral annular plane systolic excursion (MAPSE) <1 cm, CHA_2_DS_2_-VASc score, and tumor markers such as carcinoembryonic antigen (CEA) and carbohydrate antigen 19-9 (CA-19-9)^32–34^. Echocardiographic parameters and initial troponin levels are also commonly utilized^35^.

Serial cardiac imaging and echocardiography allow monitoring of left ventricular dysfunction and thrombus formation^36^. Oral anticoagulation, preferably with warfarin targeting an international normalized ratio (INR) of 2 to 3, is indicated in the presence of intracardiac thrombus for 2 to 3 months or until wall motion normalizes or thrombus resolution occurs, and in cases of atrial fibrillation^36^.

Some authors propose that patients with apical ballooning and elevated admission troponin I levels (>10 ng/mL) are at high risk for thromboembolic events and should be considered for oral anticoagulation, whereas anticoagulation should not be initiated in lower-risk patients^35^. In the present case, anticoagulation was not indicated given the absence of intracardiac thrombus and relatively preserved ejection fraction.

The complexity of TTS management and its associated thromboembolic risk necessitates a multidisciplinary approach involving cardiologists and neurologists. This collaboration is particularly critical when evaluating ischemic versus hemorrhagic risk in patients being considered for anticoagulation.

The prognosis of TTS warrants further investigation through long-term studies incorporating both cardiac and neurological follow-up. While cardiac function typically recovers within weeks, recurrence occurs in approximately 4% of cases^37^. Neurological outcomes depend primarily on initial stroke severity and frequently require rehabilitation. Long-term multidisciplinary follow-up, including cognitive assessment and psychosocial support, is essential for optimal patient care.

## Conclusion

In conclusion, TTS is a significant and often under-recognized condition characterized by reversible left ventricular dysfunction. Its relationship with ischemic stroke adds complexity, as stroke may serve as either a precipitating factor or a complication, necessitating careful neurological evaluation. Management lacks standardized guidelines; therefore, treatment must be individualized based on patient comorbidities, precipitating stressors, and complications. Further research is needed to elucidate underlying mechanisms, improve diagnostic accuracy, and establish evidence-based therapeutic strategies to optimize both neurological and cardiovascular outcomes.

**Ethics approval and consent to participate:** Patient consent was obtained.

**Consent for publication:** Patient consent was obtained.
